# The Impact of Glucagon-Like Peptide-1 (GLP-1) Agonists on Acne, Hidradenitis, and Sebaceous Activity

**DOI:** 10.7759/cureus.101212

**Published:** 2026-01-10

**Authors:** Azra Jabin, Shahab Khan, Hammad Khan, Zain Ullah Durrani, Khizer Hamza, Faiza Gul, Sana Atta Ullah, Fahad Dayam, Muhammad Yaseen, Saleem Shah

**Affiliations:** 1 Internal Medicine, Medical Teaching Institute, Lady Reading Hospital, Peshawar, PAK; 2 Acute Medicine, Portsmouth Hospitals University NHS Trust, Portsmouth, GBR; 3 Cardiology, Hayatabad Medical Complex, Peshawar, PAK; 4 Internal Medicine, Saidu Teaching Hospital, Swat, PAK; 5 Internal Medicine, Naas General Hospital, Naas, IRL; 6 Nephrology, Balochistan Institute of Nephrology and Urology, Quetta, PAK; 7 Pathology, Gajju Khan Medical College Bacha Khan Medical Complex MTI, Swabi, PAK; 8 Internal Medicine, Hayatabad Medical Complex, Peshawar, PAK; 9 Internal Medicine, Khalifa Gul Nawaz Medical And Teaching Hospital, Peshawar, PAK; 10 Internal Medicine, Peshawar Medical College, Peshawar, PAK; 11 General Medicine, Mardan Medical Complex, Mardan, PAK

**Keywords:** acne, dermatology, glp-1 receptor agonists, hidradenitis suppurativa, metabolic improvement, sebaceous gland activity, semaglutide

## Abstract

Background and objective

Glucagon-like peptide-1 receptor agonists (GLP-1 RAs), especially semaglutide, are commonly used to treat obesity and diabetes. They may influence sebaceous gland activity, hidradenitis suppurativa (HS), and acne via metabolic and anti-inflammatory pathways. This study aimed to assess the effects of semaglutide on acne severity, HS activity, and sebaceous gland function, and to evaluate associations with metabolic improvements.

Materials and methods

This prospective, observational, single-arm study was conducted at the Department of Dermatology, Hayatabad Medical Complex, Peshawar, from January 2023 to December 2024. Adults with acne, HS, or increased sebaceous gland activity who initiated semaglutide therapy between the ages of 18 and 65 years were included. Sebumetry, HS severity using the Hidradenitis Suppurativa Area and Severity Index - Revised (HASI-R), and acne grading using the Investigator’s Global Assessment (IGA) were evaluated at baseline and at three, six, 12, 18, and 24 months. Concurrent measurements of metabolic markers, including BMI, HbA1c, fasting glucose, and insulin, were also obtained. Statistical analyses included Pearson and Spearman correlations, multivariate regression to adjust for confounders, and paired t-tests for pre- and post-treatment comparisons. P-values below 0.05 were considered statistically significant.

Results

Of the 120 enrolled participants, 110 completed the follow-up (91.7%). Over 24 months, acne severity decreased from 1.92 ± 0.78 to 1.21 ± 0.63, HS activity declined from 11.34 ± 4.56 to 7.45 ± 3.21, and sebaceous gland activity was reduced from 186.45 ± 52.34 to 138.56 ± 42.78 µg/cm². Improvements in BMI, HbA1c, fasting glucose, and insulin were significantly associated with dermatologic improvement (p < 0.05). Adverse events were mild and transient and occurred in 17 participants (15.45%).

Conclusions

Semaglutide therapy was significantly associated with improvement in acne, HS activity, and sebaceous gland function, independently correlated with metabolic enhancements. These findings indicate a potential dermatologic benefit of GLP-1 agonists, supporting the need for further controlled studies.

## Introduction

Glucagon-like peptide-1 receptor agonists (GLP-1 RAs), particularly semaglutide, have reshaped the therapeutic landscape for type 2 diabetes mellitus and obesity because of their potent effects on glycemic control, weight reduction, and cardiometabolic risk modulation [1,2]. Growing evidence suggests that these agents may exert clinically significant effects beyond metabolic pathways as their use continues to expand across diverse patient populations [3]. Changes in cutaneous physiology, particularly in acne, hidradenitis suppurativa (HS), and sebaceous gland activity, have increasingly attracted attention among these potential extra-metabolic effects [4].

The function of the skin, an organ that responds to hormones, is intimately related to inflammatory and metabolic processes [5]. The pathophysiology of acne and HS is influenced by hyperinsulinemia, androgen excess, and systemic inflammation [6]. In theory, GLP-1 agonists may affect these dermatologic disorders by enhancing insulin sensitivity, decreasing circulating inflammatory mediators, and promoting substantial weight reduction [7]. Early clinical observations and case reports show variable results, with some patients reporting reductions in acne severity or HS flare-ups, while others describe new or worsened symptoms during therapy. These discrepancies underscore the need to clarify the true dermatologic effects of GLP-1 agonists and the underlying mechanisms [8].

Semaglutide may affect sebaceous gland function and cutaneous immune responses due to its strong metabolic effects, which include reduced lipogenesis, appetite suppression, and modulation of adipose-derived inflammatory pathways [9,10]. Furthermore, GLP-1 receptors are expressed in tissues linked to the pathophysiology of acne and HS, which may allow direct effects on follicular inflammation and sebaceous activity [11,12]. However, the currently available research remains limited, fragmented, and mostly observational. There remains a lack of controlled evidence investigating the relationship between GLP-1 agonist therapy and inflammatory skin conditions, and the molecular mechanisms remain uncertain.

It is both urgent and clinically important to understand the dermatologic effects of semaglutide and similar medications, given their increasing global use. A more precise characterization of their role in modifying hidradenitis, acne, and sebaceous gland activity may assist clinicians in patient selection, counseling, and treatment planning, particularly for individuals with concurrent metabolic and dermatologic conditions. This study aimed to assess the effects of GLP-1 RAs, particularly semaglutide, on acne severity, HS activity, and sebaceous gland function, and to evaluate potential associations between metabolic improvements and dermatologic outcomes.

## Materials and methods

Study design and setting

**Figure 1:**
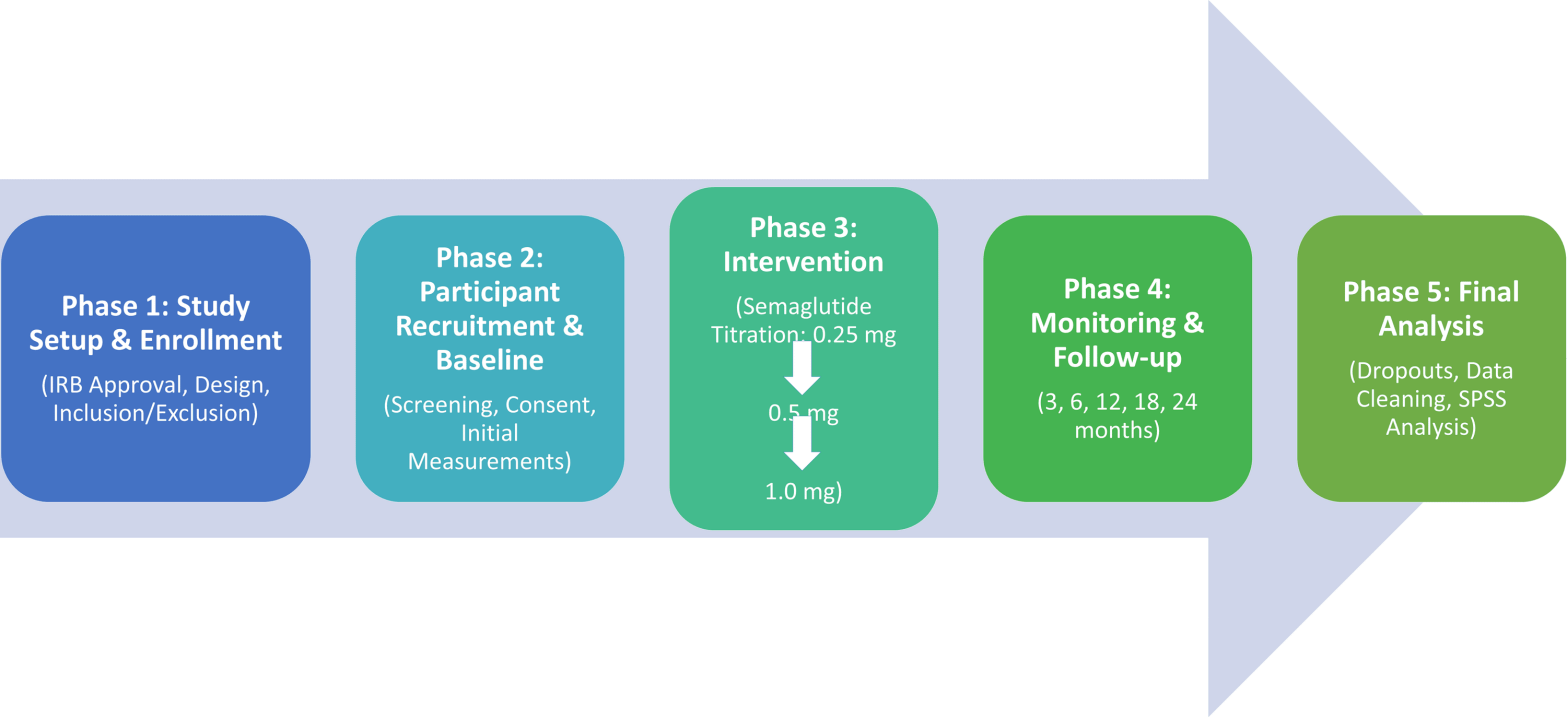
Flow diagram depicting study design, participant enrollment, and semaglutide titration protocol

This study was conducted as a prospective, observational, single-arm clinical trial at the Hayatabad Medical Complex, Peshawar. The dermatologic effects of GLP-1 RAs, specifically semaglutide, on sebaceous gland function, HS activity, and acne severity were evaluated in this research. Data were collected between January 2023 and December 2024, covering two years. The sequential process of participant recruitment, intervention titration, and longitudinal follow-up is illustrated in Figure 1.

Inclusion and exclusion criteria

The study included adults aged 18 to 65 years who were initiating GLP-1 receptor agonist therapy (semaglutide), had acne, HS, or quantifiable sebaceous gland activity (measured by sebumetry in µg/cm²), were willing to attend all scheduled follow-up visits, and could provide informed written consent. Patients who were pregnant or breastfeeding, had a known hypersensitivity or contraindication to GLP-1 RAs, had severe systemic illness or were unable to comply with follow-up, were taking systemic retinoids, hormonal therapy, or immunosuppressive medications initiated within the last three months, or had used GLP-1 agonists within the previous six months were excluded.

Sample size

Using the WHO formula for estimating a population proportion with a 95% confidence level (CI) (Z = 1.96), an expected outcome proportion of 50% (to maximize sample size in the absence of prior data), and a 10% margin of error, the initial calculation yielded 96 participants.

$n = \frac{Z^{2} \times p \times (1 - p)}{d^{2}}$

$n = \frac{(1.96)^{2} \times 0.5 \times (1 - 0.5)}{(0.10)^{2}} = 96$

To account for an anticipated 20% dropout rate and potential missing data over the 24-month follow-up, the sample size was increased to approximately 120, balancing feasibility with the need for sufficient statistical power to detect meaningful associations between GLP-1 agonist therapy and dermatologic outcomes. During the study, 10 patients were lost to follow-up and incomplete data, leaving 110 participants for the final analysis.

Dosage

Semaglutide (Ozempic®) was administered subcutaneously to participants once a week according to FDA-approved titration guidelines [13]. The initial dose was 0.25 mg/week for four weeks, followed by 0.5 mg/week, with up-titration to 1.0 mg/week based on glucose control and tolerability. If clinically indicated, the dose could be further increased to 2.0 mg/week. At each follow-up visit, medication adherence was monitored, and any adverse events, missed doses, or dose adjustments were documented.

Data collection

Baseline data included demographic information, medical history, metabolic profile (BMI, fasting glucose, insulin, HbA1c, and lipid panel), and dermatologic status. At baseline and all follow-up visits, potential confounding variables were thoroughly documented, including dietary habits, skincare routines, use of topical or over-the-counter products, and physical activity levels. Clinical evaluations were consistently conducted at baseline and at three, six, 12, 18, and 24 months.

Acne severity was evaluated using the validated Investigator’s Global Assessment (IGA) [14], along with a structured 5-point ordinal grading system ranging from 0 (clear) to 4 (severe). Grading considered overall disease severity and lesion characteristics, incorporating standardized assessment of inflammatory lesions (papules, pustules, and nodules) and non-inflammatory lesions (open and closed comedones) across predefined facial and truncal regions. This structured lesion assessment supported severity classification and ensured consistency across visits and longitudinal comparisons, rather than serving as an independent outcome measure.

HS activity was measured using the International Hidradenitis Suppurativa Severity Score System (IHS4) [15], a publicly available and validated tool that evaluates disease severity based on standardized counting of inflammatory nodules, abscesses, and draining tunnels across affected anatomical locations, allowing objective tracking of inflammation over time.

Sebaceous gland function was assessed quantitatively using sebumetry at predetermined facial and truncal sites, with measurements expressed in µg/cm² under standardized conditions. At each visit, medication adherence, side effects, and metabolic changes during semaglutide treatment were systematically documented. The specific clinical criteria and score thresholds used to categorize disease severity for both acne and HS are presented in Table 1.

**Table 1:** Clinical grading criteria for acne and HS) HS: hidradenitis suppurativa; IGA: Investigator’s Global Assessment; HASI-R: Hidradenitis Suppurativa Area and Severity Index - Revised; IHS4: International Hidradenitis Suppurativa Severity Score System

Condition	Assessment tool	Grade/score	Clinical description
Acne	IGA scale	Mild (0–1)	Clear to almost clear; rare non-inflammatory lesions (comedones) and very few papules
		Moderate (2)	Many non-inflammatory lesions; several inflammatory lesions (papules/pustules); no nodules
		Severe (3)	Numerous inflammatory and non-inflammatory lesions; presence of inflammatory nodules
HS	HASI-R/IHS4	Mild	Score ≤3: isolated nodules/abscesses without evidence of fistulae (tunnels)
		Moderate	Score 4–10: multiple nodules/abscesses and limited skin tunneling
		Severe	Score ≥11: diffuse involvement with multiple interconnected tunnels and abscesses
Sebaceous gland	Sebumetry	µg/cm^2^	Quantitative measurement of skin surface lipids under standardized conditions

Statistical Analysis

SPSS Statistics version 26 (IBM Corp., Armonk, NY) was used to analyze the data. Categorical data were shown as frequencies and percentages, whilst continuous variables were given as mean ± standard deviation (SD). Depending on normality, paired t-tests were used for pre-post comparisons. Pearson or Spearman correlation coefficients were used to evaluate relationships between metabolic improvement and dermatological results. To account for possible confounders, such as age, sex, BMI, baseline metabolic parameters (HbA1c, fasting glucose, lipid profile), dietary practices, skincare regimens, physical activity, and concurrent drugs that may affect dermatological results, multivariate analyses were performed. P-values less than 0.05 were considered statistically significant.

Ethical approval

The study was reviewed and approved by the Institutional Review Board (IRB) of Hayatabad Medical Complex, Peshawar. (Approval No. 761/CD/HMC/2022, Dated: 9/12/2022). Written informed consent was obtained from all participants before enrollment, ensuring confidentiality and adherence to the Declaration of Helsinki.

## Results

The study included 110 participants, comprising 51 males (46.36%) and 59 females (53.64%), with a mean age of 42.15 ± 11.23 years (Table 2). Dermatologically, acne severity was mild in 35 participants (31.82%), moderate in 50 (45.45%), and severe in 25 (22.73%). HS activity was mild in 38 participants (34.55%), moderate in 50 (45.45%), and severe in 22 (20.00%). All participants were adherent to therapy at baseline (110/110, 100%).

**Table 2:** Baseline characteristics of study participants (n = 110) BMI: body mass index; HbA1c: hemoglobin A1c; LDL: low-density lipoprotein; HDL: high-density lipoprotein; IGA: Investigator’s Global Assessment; HS: hidradenitis suppurativa; HASI-R: Hidradenitis Suppurativa Area and Severity Index – Revised; SD: standard deviation

Category		Variable	Value
Demographics	Age (years)	Mean ± SD	42.15 ± 11.23
	Gender	Male	51 (46.36%)
		Female	59 (53.64%)
Metabolic profile	BMI (kg/m²)	Mean ± SD	31.28 ± 5.14
	HbA1c (%)	Mean ± SD	7.92 ± 1.34
	Fasting glucose (mg/dL)	Mean ± SD	138.45 ± 28.62
	Fasting insulin (µIU/mL)	Mean ± SD	16.45 ± 5.12
	Lipid profile (mg/dL), mean ± SD	Total cholesterol	198.12 ± 34.56
		LDL	118.23 ± 27.34
		HDL	43.56 ± 8.21
		Triglycerides	156.45 ± 42.12
Dermatologic status	Acne severity (IGA), n (%)	Mild (0–1)	35 (31.82%)
		Moderate (2)	50 (45.45%)
		Severe (3)	25 (22.73%)
	HS activity (HASI-R), n (%)	Mild	38 (34.55%)
		Moderate	50 (45.45%)
		Severe	22 (20.00%)
	Sebaceous gland activity (µg/cm²)	Mean ± SD	186.45 ± 52.34
Medication adherence		N (%)	110 (100.00%)

Over the 24-month follow-up, acne severity (IGA) decreased progressively from 1.92 ± 0.78 at baseline among 110 participants (100%) to 1.21 ± 0.63 at 24 months among 96 participants (87.27%). HS activity (HASI-R) decreased from 11.34 ± 4.56 in 110 participants (100%) to 7.45 ± 3.21 in 96 participants (87.27%), while sebaceous gland activity declined from 186.45 ± 52.34 µg/cm² to 138.56 ± 42.78 µg/cm² over the same period (Table 3). Medication adherence decreased from 110 participants (100%) at baseline to 96 participants (87.27%) at 24 months due to missed doses.

**Table 3:** Dermatologic outcomes during semaglutide therapy (n = 110) Medication adherence decreased over time due to missed doses. Values represent the number of participants completing follow-up at each time point. Ten participants were lost to follow-up and excluded from analysis, so adherence reflects actual adherence among the remaining 110 participants IGA: Investigator Global Assessment; HS: hidradenitis suppurativa; HASI-R: Hidradenitis Suppurativa Area and Severity Index – Revised; SD: standard deviation

Timepoint	Acne severity (IGA), mean ± SD	HS activity (HASI-R), mean ± SD	Sebaceous gland activity (µg/cm²), mean ± SD	Medication adherence, n (%)
Baseline	1.92 ± 0.78	11.34 ± 4.56	186.45 ± 52.34	110 (100.00%)
3 months	1.85 ± 0.75	10.78 ± 4.42	178.56 ± 50.12	107 (97.27%)
6 months	1.72 ± 0.71	9.98 ± 4.12	170.23 ± 48.56	105 (95.45%)
12 months	1.56 ± 0.69	9.02 ± 3.78	162.78 ± 47.12	102 (92.73%)
18 months	1.36 ± 0.65	8.12 ± 3.45	150.45 ± 45.67	99 (90.00%)
24 months	1.21 ± 0.63	7.45 ± 3.21	138.56 ± 42.78	96 (87.27%)

**Figure 2:**
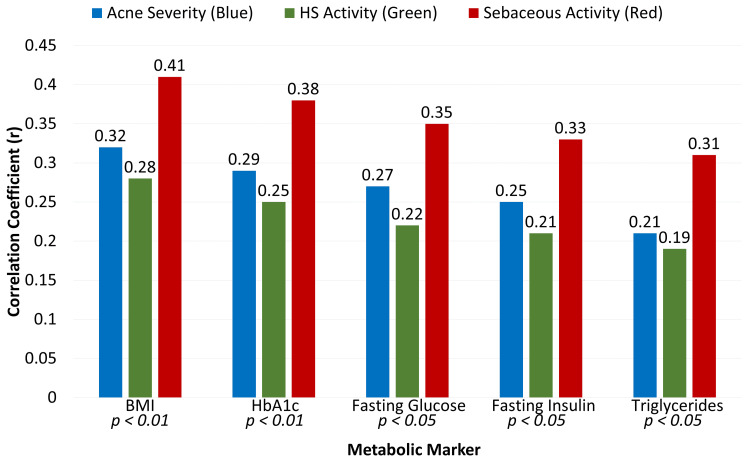
Correlation between metabolic improvement and dermatologic outcomes at 24 months (n = 110) P-values < 0.05 were significant BMI: body mass index; HbA1c: hemoglobin A1c; HS: hidradenitis suppurativa

At 24 months, reductions in BMI, HbA1c, fasting glucose, fasting insulin, and triglycerides showed positive correlations with improvements in acne severity, HS activity, and sebaceous gland activity (Figure 2). Correlation coefficients ranged from 0.19 to 0.41, with all associations statistically significant (p < 0.05), underscoring a link between metabolic improvement and favorable dermatologic outcomes during GLP-1 agonist therapy.

**Figure 3:**
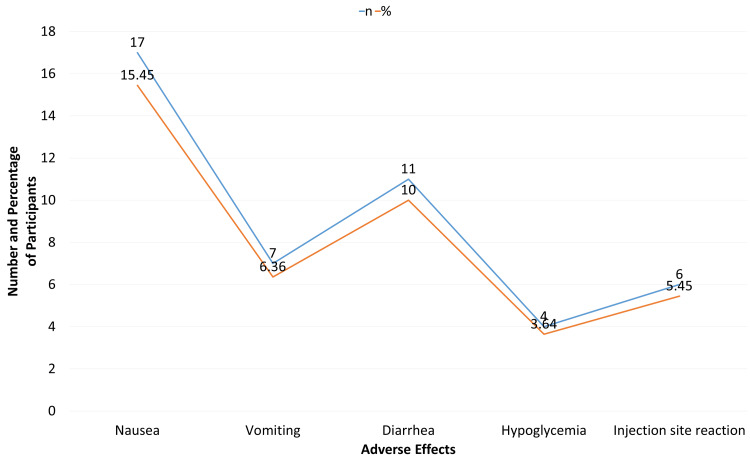
Adverse events during semaglutide therapy (n = 110)

Adverse events were generally mild and included nausea in 17 participants (15.45%), vomiting in seven (6.36%), diarrhea in 11 (10.00%), hypoglycemia in four (3.64%), and injection site reactions in six (5.45%) (Figure 3).

After adjusting for confounders, improvements in BMI, HbA1c, fasting glucose, and insulin were significantly associated with reductions in acne severity, HS activity, and sebaceous gland activity (adjusted β range: 0.15-0.35, p < 0.01), as shown in Table 4. Age and sex were not significant predictors. These results confirm that metabolic improvements independently contribute to dermatologic benefits during semaglutide therapy.

**Table 4:** Multivariate analysis: adjusted associations between metabolic improvement and dermatologic outcomes at 24 months (n = 110) Adjusted β represents the change in dermatologic outcome per unit change in metabolic variable, controlling for confounders: age, sex, baseline BMI, baseline HbA1c, fasting glucose, lipid profile, dietary habits, skincare routines, physical activity, and concomitant medications. Δ = change from baseline to 24 months OR: odds ratio; CI: confidence interval; IGA: Investigator’s Global Assessment; HS: hidradenitis suppurativa; HASI-R: Hidradenitis Suppurativa Area and Severity Index – Revised; BMI: body mass index; HbA1c: hemoglobin A1c

Dermatologic outcome	Independent variable	Adjusted β/OR	95% CI	P-value
Acne severity (IGA)	ΔBMI	0.28	0.12 – 0.44	<0.01
	ΔHbA1c	0.24	0.08 – 0.40	0.004
	ΔFasting glucose	0.21	0.05 – 0.37	0.01
	ΔInsulin	0.18	0.03 – 0.33	0.02
	Age	0.05	-0.02 – 0.12	0.14
	Sex (male vs. female)	0.03	-0.11 – 0.17	0.65
HS activity (HASI-R)	ΔBMI	0.25	0.10 – 0.40	0.002
	ΔHbA1c	0.20	0.06 – 0.34	0.005
	ΔFasting glucose	0.18	0.04 – 0.32	0.01
	ΔInsulin	0.15	0.01 – 0.29	0.03
	Age	0.04	-0.03 – 0.11	0.21
	Sex (male vs. female)	0.02	-0.12 – 0.16	0.75
Sebaceous gland activity (µg/cm²)	ΔBMI	0.35	0.20 – 0.50	<0.001
	ΔHbA1c	0.30	0.16 – 0.44	<0.001
	ΔFasting glucose	0.27	0.12 – 0.42	0.001
	ΔInsulin	0.23	0.09 – 0.37	0.002
	Age	0.06	-0.01 – 0.13	0.09
	Sex (male vs. female)	0.04	-0.10 – 0.18	0.57

## Discussion

Our results, which demonstrate that semaglutide gradually reduces acne severity, HS activity, and sebaceous gland activity over 24 months, are consistent with and extend growing evidence that GLP-1 RAs may be beneficial for inflammatory skin disorders. According to a recent comprehensive review, GLP-1 RAs such as semaglutide and liraglutide have been shown to reduce systemic inflammation and the severity of HS lesions. One possible explanation for this effect is the suppression of pro-inflammatory cytokines such as TNF-α and IL-17 [16]. GLP-1 modulation represents a potential adjuvant in the treatment of HS, and the steady decline in HS activity in our cohort over time offers empirical longitudinal support for this anti-inflammatory theory.

The literature on GLP-1 RAs and acne is more diverse than that of HS. Semaglutide was one of many GLP-1 treatments included in a meta-analysis that found no conclusive evidence of an acne-promoting impact directly related to the medications. Instead, the authors hypothesized that changes in acne during therapy are likely caused by hormonal changes brought on by weight reduction [17]. Semaglutide may be linked to net dermatologic improvement rather than aggravation, at least in our sample, based on our finding that acne improved gradually (mean IGA decreased from ~1.9 to ~1.2). This might be due to variations in patient characteristics, the severity of acne at baseline, or metabolic reactions (e.g., insulin alterations, weight loss).

The wider dermatologic safety profile of GLP-1 RAs should be taken into account when evaluating therapy cessation and adherence. A comprehensive analysis of hundreds of studies identified both positive outcomes, such as reduced HS flares and inflammation, and negative skin responses, such as injection-site reactions and urticaria [18]. Our low rate of severe adverse events and high adherence reflect the overall positive tolerability reported in the literature, even though our study did not focus on uncommon immune-mediated skin responses.

Comparing our results with cohort-level epidemiologic data helps place them in context. According to a recent multicenter TriNetX cohort study, compared with matched controls, patients with HS who received GLP-1 RAs had significantly fewer hospitalizations (hazard ratio (HR): 0.87) and procedures linked to HS (HR: 0.35) [19]. Although hospitalizations and surgical interventions were not included in our study, the reduction in disease activity we report likely contributes to these positive hard-endpoint outcomes observed in larger populations.

Furthermore, limited interventional investigations using different GLP-1 agonists provide mechanistic support for our clinical findings. For example, a pilot research with liraglutide in obese HS patients showed significant decreases in BMI, CRP, and the severity of HS lesions (Hurley staging), but the improvements in inflammation were not fully explained by weight loss [20]. In line with our multivariate analyses, which showed that metabolic changes (e.g., ΔBMI, ΔHbA1c) remained strongly linked with dermatologic outcomes even after controlling for covariates, these findings imply that GLP-1-mediated immunomodulation functions beyond simple metabolic improvement.

However, there are still important unanswered questions. Recent pharmacovigilance and registry data, for instance, indicate that a greater number of dermatologic events, including injection-site reactions, alopecia, and even less common conditions such as "Ozempic face" (facial fat loss), are being reported with GLP-1 RAs. This emphasizes the need for targeted safety monitoring [21]. Systematic studies also underscore the need for biomarkers to predict dermatologic improvement, noting that while anti-inflammatory effects are encouraging, individual patient responses vary considerably [18].

Overall, this study adds valuable prospective evidence indicating that semaglutide may safely and significantly improve sebaceous function, HS activity, and acne over two years. These findings reinforce previous studies while also highlighting the therapeutic potential of GLP-1 RAs in dermatology and the need for ongoing attention to cutaneous safety and mechanism-based stratification of patients.

Strengths and limitations

This research offers several noteworthy advantages. Compared with cross-sectional or short-term studies, it is among the few prospective, longitudinal investigations to assess the dermatologic effects of semaglutide over two years, allowing for a more accurate evaluation of changes over time. The use of objective measures, such as standardized acne grading, validated hidradenitis suppurativa scoring, and quantitative sebumetry, enhances methodological rigor and minimizes subjective bias. Comprehensive metabolic profiling supports robust correlation and multivariate analyses that clarify independent relationships between metabolic improvements and dermatologic outcomes. Internal validity is further strengthened through regular follow-up and adherence monitoring.

This study has several limitations. The single-arm observational design and lack of a control group limit causal inference and make it difficult to distinguish drug-related effects from natural disease fluctuations or lifestyle changes. Conducting the study at a single center may also restrict generalizability to broader populations with different demographic characteristics or comorbid conditions. Although statistical adjustments were made for confounders, residual confounding from unmeasured factors such as hormonal fluctuations, unreported use of topical therapies, or differences in the skin microbiota cannot be fully excluded. In addition, while the sample size is adequate for the primary outcomes, it may be insufficient to detect rare dermatologic adverse events. Further multicenter randomized controlled trials are needed to confirm these findings and to better elucidate the mechanisms underlying GLP-1-mediated improvements in skin disease.

## Conclusions

Over a 24-month period, treatment with semaglutide was associated with significant and sustained improvements in sebaceous gland function, hidradenitis suppurativa activity, and acne severity, along with marked metabolic benefits. These findings suggest that GLP-1 receptor agonists may offer therapeutic effects beyond glycemic control and weight reduction by positively influencing sebaceous activity and cutaneous inflammation, particularly in patients with concurrent metabolic and dermatologic conditions. Although this study contributes to the growing evidence supporting a dermatologic role for GLP-1-based therapies, larger controlled trials are needed to confirm these observations, clarify underlying mechanisms, and inform their integration into routine dermatologic practice.
